# Successful treatment of arrhythmia-induced cardiomyopathy in an infant with tuberous sclerosis complex

**DOI:** 10.1186/s12887-016-0557-2

**Published:** 2016-01-25

**Authors:** Noriko Motoki, Yuji Inaba, Satoshi Matsuzaki, Yohei Akazawa, Takafumi Nishimura, Tetsuhiro Fukuyama, Kenichi Koike

**Affiliations:** Department of Pediatrics, Shinshu University School of Medicine, Asahi 3-1-1, Matsumoto, 390-8621 Japan

**Keywords:** Tuberous sclerosis complex, Cardiac rhabdomyoma, Dilated cardiomyopathy, Arrhythmia, Carvedilol

## Abstract

**Background:**

Tuberous sclerosis complex (TSC) is an autosomal-dominant tumor suppressor gene syndrome that is characterized by the development of distinctive benign tumors and malformations in multiple organ systems (N Eng J Med 355:1345-1356, 2006). Cardiac rhabdomyomas are intracavitary or intramural tumors observed in 50–70 % of infants with TSC but only cause serious clinical problems in a very small fraction of these patients (N Eng J Med 355:1345-1356, 2006; Pediatrics 118:1146-1151, 2006; Eur J Pediatr 153:155-7, 1994); most individuals have no clinical symptoms and their tumors spontaneously regress. However, despite being clinically silent, these lesions can provoke arrhythmias and heart failure (Pediatrics 118:1146-1151, 2006; Eur J Pediatr 153:155-7, 1994).

**Case presentation:**

We here report the clinical findings of an infant suffering from TSC complicated with dilated cardiomyopathy (DCM) after the regression of cardiac rhabdomyomas. Although his tumors improved spontaneously, tachycardia and irregular heart rate due to frequent premature ventricular and supraventricular contractions persisted from the newborn period and were refractory to several medications. His cardiomyopathy was suspected to have been induced by the tachycardia or arrhythmia. We found carvedilol therapy to be safe and highly effective in treating the cardiomyopathy. To our knowledge, this is the first case report of TSC with DCM after regression of cardiac tumors and its successful treatment.

**Conclusion:**

The patient’s clinical course suggests that careful life-long disease management is important, even in TSC patients without apparent symptoms.

## Background

Tuberous sclerosis complex (TSC) is an autosomal dominant tumor suppressor gene syndrome that is characterized by the development of benign tumors and malformations in multiple organ systems [1]. Although cardiac rhabdomyomas are observed in approximately 50–70 % of infants with TSC, most patients exhibit no clinical symptoms and their tumors spontaneously regress [1–3]. However, such tumors occasionally cause serious clinical problems, including symptomatic cardiac failure and several kinds of arrhythmia [2, 3]. We here report the successful treatment with carvedilol of an infant with TSC complicated with dilated cardiomyopathy (DCM) which might have been induced by arrhythmia after regression of cardiac rhabdomyomas.

## Case presentation

**Fig. 1:**
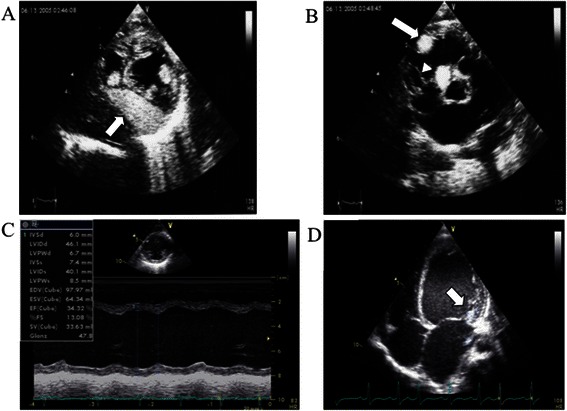
Echocardiographic findings. At birth, tumors were located on the postero-septal wall of the left ventricle (**a**, *arrow*), right ventricular outflow (**b**, *arrow*), and intraventricular septum (**b**, *arrowhead*) at birth. At the age of 23 months, 2D echocardiography disclosed an enlarged left ventricle (**c**), and M-mode echocardiography revealed poor left ventricular wall contraction (**d**)

**Fig. 2:**
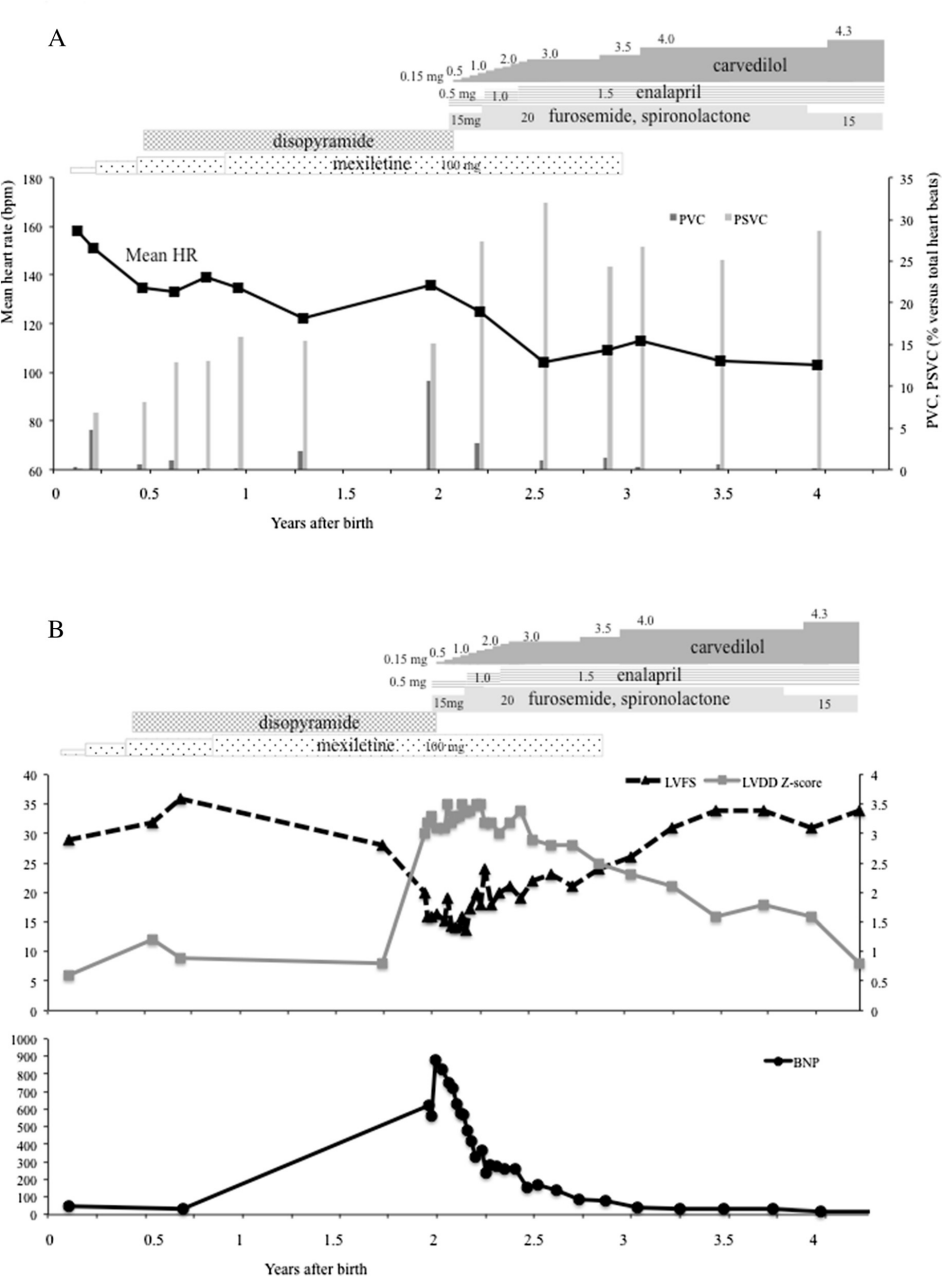
**a**, **b** Changes in cardiac function before and during treatment with carvedilol. BNP, Brain natriuretic peptide; LVDd, Left ventricular end-diastolic diameter; LVFS, Left ventricular fractional shortening; HR: Heart rate; PSVC, Premature supraventricular contraction; PVC, Premature ventricular contraction

The male patient was born spontaneously at term after an uneventful pregnancy despite the detection of cardiac tumors at 36 weeks by fetal echocardiography. After birth, he was diagnosed as having TSC based on the diagnostic criteria of white leaf-shaped hypomelanotic macules, a subependymal nodule, and a retinal hamartoma [4]. Multiple intramural cardiac tumors of 5–25 mm in diameter were found by transthoracic echocardiography (Fig. 1a, b). Soon after birth, the boy exhibited numerous episodes of premature supraventricular contractions (PSVC), multifocal premature ventricular contractions (PVC), and non-sustained ventricular tachycardia (NSVT). Mexiletine treatment was commenced at 1 month of age (Fig. 2a). Since a maximum NSVT of 14 beats at a rate of 269 bpm was detected by Holter electrocardiography (ECG) at the age of 5 months, we added disopyramide to his therapy regime, which resulted in a decline in NSVT frequency. The tumors gradually diminished in size, but his frequent PSVC and tachycardia persisted.

At 20 months of age, the patient’s cardiac function was preserved; echocardiography revealed a left ventricular end-diastolic diameter (LVDd) of 30.8 mm (Z-score: 0.5) and a left ventricular fractional shortening (LVFS) of 28 %.

**Fig. 3:**
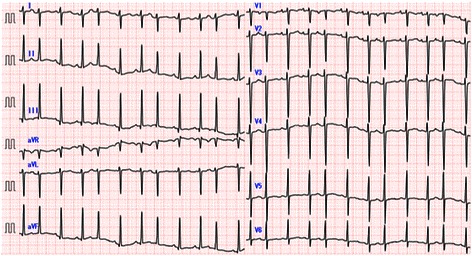
Electrocardiography findings. Twelve-lead electrocardiography (ECG) revealed irregular tachycardia (139 bpm) and sporadic PSVC without ST abnormality or wide QRS duration

At 23 months of age, he was admitted to our hospital due to cardiac dysfunction. His body height and weight were 85 cm (+0.1 SD) and 11.5 kg (+0.1 SD), respectively. Blood pressure was 88/47 mmHg. Heartbeat was irregular at 134 bpm without extra heart sounds or murmurs. Neither hepatomegaly nor pretibial edema was observed. Laboratory data including blood cell counts, electrolytes, CRP, CK-MB, troponin T, glucose, ammonia, lactic and pyruvic acid, and thyroid function showed no abnormalities. However, brain natriuretic peptide (BNP) and human atrial natriuretic peptide levels were markedly increased at 619 pg/ml (normal range: < 20 pg/ml) and 654 pg/ml (normal range: < 43 pg/ml), respectively. There were no significant increases of viral antibody titers in his serum, including those for the Coxsackie virus, adenovirus, or echovirus. His cardiothoracic ratio was 64 % on a chest radiograph. Twelve-lead ECG revealed irregular tachycardia (139 bpm) and sporadic PSVC without ST abnormality or wide QRS duration (Fig. 3). According to Holter ECG, the patient’s total number of heartbeats (THB) was 195,807 per day and mean heart rate was 136 bpm. Frequent multifocal PVC (20,883 beats per day; 10.7 % of THB) and PSVC (29,733 beats per day; 15.2 % of THB) were also noted. Echocardiography revealed cardiac dilatation and diffuse hypokinesis, LVDd of 45.2 mm (Z-score: 3.0), and LVFS of 15 % (Fig. 1c). Severe mitral insufficiency caused by the expansion of the mitral annulus was detected as well. His coronary arteries were normal. Surprisingly, his cardiac tumors had diminished in size and were recognizable as a small bright area in the posterior mitral valve (Fig. 1d).

Diuretics and angiotensin converting enzyme inhibitor were administered as anti-heart failure therapies. To control tachycardia and reduce afterload on the failing heart, we also commenced oral administration of carvedilol at 0.15 mg/day (0.01 mg/kg/day) that was gradually increased to 3.0 mg/day (0.3 mg/kg/day) 3 months later. Although frequent PSVC persisted, the boy’s mean HR gradually decreased and the incidence of PVC and its short run became markedly diminished (Fig. 2a). BNP gradually decreased to within normal range. The abnormal values of LVFS and LVDd persisted for 1 year after the initiation of carvedilol treatment, but both parameters eventually improved to normal levels (34 % and 35.8 mm [Z-score: 0.8], respectively) by the age of 4 years (Fig. 2b).

## Discussion

We here report the clinical findings of an infant boy suffering from TSC complicated with DCM. Although his tumors regressed spontaneously, tachycardia and heart rate irregularity due to frequent PSVC and PVC persisted from the newborn period. After the commencement of carvedilol aimed to control heart rate and reduce afterload in the failing heart, BNP and LV systolic function improved within months and LV volume recovered slowly over several years.

The most frequent cardiac involvement in TSC is multiple cardiac rhabdomyomas, which may spontaneously regress during the first few years. However, TSC is a known cause of heart failure because of myocardial depression and inflow or outflow obstruction by tumors [1–3]. TSC is also classified as one of the secondary causes of cardiomyopathy [5]. Several reports have described DCM as a primary cardiac involvement in patients with TSC [5, 6]. Although 1 case was of TSC and DCM without cardiac tumors, the cause of the cardiomyopathy was unknown [6]. Our patient’s cardiac tumors had improved spontaneously by the time of the appearance of cardiac dysfunction. Differential diagnoses for his heart failure included myocarditis, coronary artery abnormality, hormone or metabolic abnormalities, and mitochondrial disease, but it was ultimately difficult to pinpoint its precise nature.

TSC-related cardiac rhabdomyomas can combine with several other kinds of arrhythmias [1–3]. However, the relationship between tachycardia/arrhythmia and cardiomyopathy has not been clearly described in TSC. Here, we consider that tachycardia or arrhythmia was responsible for the patient’s cardiomyopathy.

Atrial ectopic tachycardia is the most common arrhythmia in pediatric arrhythmia-induced cardiomyopathy (AIC), for which beta-blockers are frequently used as the first-line therapy [7]. Heart rate irregularity also occurs in pediatric AIC as salvos of tachycardia interspersed with periods of sinus rhythm [8]. Prior reports have described weeks to months for LV systolic functional recovery in AIC, and months to years for reverse remodeling to bring heart rate and rhythm under control [9, 10].

The relationship between arrhythmia and cardiomyopathy can be difficult to characterize because arrhythmias may exist for years before their recognition and the development of cardiomyopathy [8]. Cardiac magnetic resonance imaging may help differentiate AIC from DCM; in many cases, patients with DCM that does not improve exhibit findings in late gadolinium enhancement suggestive of an underlying scar [11].

As a non-selective beta-blocker with additional vasodilating properties, carvedilol has been demonstrated to improve survival in adult patients with severe chronic heart failure [12]. In a multicenter, randomized, double-blind, placebo-controlled trial by Shaddy et al. [13], carvedilol did not significantly affect the primary end point of composite clinical outcomes in children or adolescents with symptomatic heart failure due to systemic ventricular dysfunction. However, a prespecified subgroup analysis noted significant interaction between treatment and ventricular morphology, indicating a possible differential effect between patients with a systemic left ventricle (i.e., beneficial trend) and those whose systemic ventricle was not the left ventricle (i.e., non-beneficial trend). Other studies have shown carvedilol to be effective for chronic heart failure in pediatric patients with systemic left ventricle, such as DCM [14, 15]. Additionally, chronic use of a beta-blocker in conjunction with ACE inhibitor therapy improved clinical status and exercise tolerance in patients with heart failure [16]. The beta-blocking effects of carvedilol may be important because catecholamines induce oxidative stress in the myocardium, and the direct antioxidant effect of this drug may contribute to oxidative stress relief in the failing myocardium [15]. Accordingly, carvedilol-mediated reductions in heart rate and oxidative stress were suspected to have accounted for the improvement in LV dysfunction in our patient. Special attention must be paid to the negative inotropic effect of carvedilol during treatment initiation in patients with severe cardiac dysfunction [14, 17]. We therefore started with a very small dose, and gradually increased it to avoid any adverse effects on cardiac function.

## Conclusion

To our knowledge, this is the first reported case of an infant suffering from TSC complicated with DCM after the regression of cardiac rhabdomyomas whose cardiomyopathy was suspectedly induced by arrhythmia. We found that carvedilol therapy was safe and effective for treating the cardiomyopathy despite the patient’s very young age. TSC rhabdomyomas generally disappear spontaneously and have a good prognosis. However, the present case exhibited heart failure with severely reduced LV function during the course of illness, suggesting the importance of meticulous life-long follow-up, even in apparently asymptomatic patients with TSC.

## Consent

Written informed consent was obtained from the patient’s parents for publication of this case report and any accompanying images. A copy of the written consent is available for review by the Editor-in-Chief of this journal.

## Abbreviations

AIC: arrhythmia-induced cardiomyopathy
BNP: brain natriuretic peptide
DCM: dilated cardiomyopathy
ECG: electrocardiography
LVDd: left ventricular end-diastolic diameter
LVFS: left ventricular fractional shortening
NSVT: non-sustained ventricular tachycardia
PSVC: premature supraventricular contractions
PVC: premature ventricular contractions
THB: total number of heartbeats
TSC: tuberous sclerosis complex
